# Obituary

**Published:** 1893-03

**Authors:** 


### Obituary.

DR. GEORGE JACKSON FISHER, of Sing Sing, one of the most prominent physicians of the State, died at his residence February 4, 1893, from blood-poisoning contracted while performing an operation on January 13, 1893. Dr. Fisher was one of the most accomplished men of his time, and had collected a large library filled with quaint and curious literature, which was often sought by literary men. He was a member of various scientific bodies, and had been President of the Medical Society of the State of New York. He was altogether one of the most gifted and charming conversationalists of the day. His demise will be mourned by a wide circle of professional and social friends throughout the country.

